# Tailored surgery for large adrenal tumors: the minimally invasive to open (hybrid) approach

**DOI:** 10.1007/s13304-025-02388-7

**Published:** 2025-08-29

**Authors:** Agata Dukaczewska, Konrad Ilgner, Catarina Alisa Kunze, Jennifer Sladek, Eva Maria Dobrindt, Peter E. Goretzki, Johann Pratschke, Martina T. Mogl, Frederike Butz

**Affiliations:** 1 Department of Surgery, Campus Charité Mitte | Campus Virchow-Klinikum, Charité – Universitätsmedizin Berlin, Corporate Member of Freie Universität Berlin, Humboldt-Universität zu Berlin, 10117 Berlin, Germany; 2 Department of Pathology, Charité – Universitätsmedizin Berlin, Corporate Member of Freie Universität Berlin, Humboldt-Universität zu Berlin, Berlin, Germany; 3 Department of Endocrine Surgery, Helios Universitätsklinikum Wuppertal, Universität Witten-Herdecke, Heusnerstraße 40, 42283 Wuppertal, Germany

**Keywords:** Adrenalectomy, Conversion, Large adrenal tumours

## Abstract

Conversion from minimally invasive techniques to open surgery has mostly been considered as an undesirable event associated with intraoperative complications or poor preoperative planning. However, the impact of conversion to open surgery during adrenalectomy for large adrenal tumours remains unclear. This study investigates the outcomes of conversion from minimally invasive to open surgery for adrenal masses ≥ 60 mm with an additional focus on the identification of survival predictors in patients with large adrenocortical carcinoma (ACC). We retrospectively analyzed 97 patients who underwent unilateral adrenalectomy for tumours ≥ 60 mm. Patient characteristics, tumour features, surgical approaches, and outcomes were compared. Survival outcomes in ACC patients (*n* = 34) were assessed using Kaplan–Meier analysis, with prognostic factors evaluated via univariate Cox regression and Ridge Regression modeling. Of 97 patients, 41 (42%) underwent minimally invasive adrenalectomy (MIA), 40 (41%) open adrenalectomy (OA), and 16 (17%) required conversion to open surgery (hybrid adrenalectomy, HA). HA had a longer operative time (median 226.5 vs. 108.5 min; p < 0.001) and hospital stay (median 9 vs. 4 days; *p* < 0.001) compared to MIA but not OA (median 188 min; *p* = 0.102; 10 days; *p* = 0.519, respectively). Overall, complications were more frequent in HA (43.7%) than MIA (7.3%; *p* = 0.003) but similar to OA (37.5%; *p* = 0.897). Minor complications were more common in HA than MIA (31 vs. 7%; *p* = 0.032), while major complications were comparable (12.5 vs. 7.5%; *p* = 0.617). Tumours in HA cases more often showed vascular infiltration (*p* = 0.001) and required multivisceral resection (*p* = 0.002). ENSAT tumour stage (OS: HR = 4.66, *p* = 0.041; PFS: HR = 2.52, *p* = 0.005) and the S-GRAS score (OS: HR = 6.00; PFS: HR = 1.50) were significant survival predictors in ACC, whereas the operative technique was not. Conversion to open surgery increases minor complications compared to MIA but not OA. ENSAT tumour stage and S-GRAS score predict survival in ACC, while the surgical approach does not. Timely conversion should be performed to ensure oncological safety when needed.

**Supplementary Information:**

The online version contains supplementary material available at 10.1007/s13304-025-02388-7.

## Introduction

While minimally invasive techniques are currently the gold standard in the treatment of non-invasive adrenal tumours measuring less than 60 mm [1–4], open adrenalectomy is recommended when malignancy and local invasion is suspected to prevent rupture of the tumor capsule [2, 5]. However, as surgical experience with minimally invasive procedures for adrenal tumours continues to grow, the indications for laparoscopic adrenalectomy have expanded [6–8]. When minimally invasive surgery is applied to large adrenal tumours, the possibility or necessity of conversion to open surgery should be evaluated. To date, conversion has mostly been seen as an undesirable event associated with intraoperative bleeding [9] and an elevated rate of R1 resections [10]. Large tumour size, histological type (pheochromocytoma or malignant tumour) and the need for multivisceral resection [6, 11, 12] were identified as risk factors for conversion at the planning stage of the operation thereby justifying an open surgical approach [11]. The implications of conversion, however, have not been sufficiently investigated. Studies on conversion are scarce and yield contradictory results [6, 9, 13]. As conversion is an important security measure that should consciously be applied to prevent undesirable intraoperative events [4], there is an urgent need to reduce bias towards it. Furthermore, a hybrid technique that involves a planned transition from minimally invasive to open surgery may be selected in specific cases to benefit from both approaches within a single operation [14].

We conducted the current study to retrospectively evaluate the impact of conversion on operative and postoperative outcomes in the surgical treatment of large adrenal tumours in patients treated at the tertiary centre for endocrine surgery of Charité - Universitätsmedizin Berlin .

## Materials and methods

### Patient demographic and clinical data

468 consecutive patients who underwent adrenalectomy from January 2008 to April 2023 at the tertiary centre of endocrine surgery of Charité - Universitätsmedizin Berlin were included in a retrospective database. Patients who underwent bilateral adrenalectomy (*n* = 12), who had prior surgery on the ipsilateral or contralateral adrenal gland (*n* = 9), or who were operated on for adrenal haematoma were excluded from the study. Finally, patients who underwent unilateral surgery for an adrenal tumour measuring at least 60 mm (*n* = 97) were retrieved from the database and assigned to three groups on the basis of the surgical technique used for adrenalectomy: minimally invasive (MIA), open (OA) and hybrid adrenalectomy (HA), the latter including conversion from a minimally invasive to an open approach. None of the tumors in this cohort were resected using a robotic technique. The abovementioned surgical approaches were compared with respect to patient-specific factors (patient age, sex, body mass index (BMI) and physical status according to American Society of Anaesthesiologists (ASA) classification), tumour-specific factors (tumour type, size, and location, presence of vascular and/or adjacent organ infiltration and lymph node metastases, hormone production), intraoperative and postoperative outcomes (duration of surgery, necessity of multivisceral resection, need for intraoperative or postoperative blood transfusion, requirement for postoperative intensive care unit (ICU) monitoring, length of stay in the ICU and postoperative hospital stay) and occurrence of postoperative complications according to the Clavien‒Dindo classification [15], with minor complications including all 1–3a complications and major complications greater than or equal to 3b. The reasons for conversion in the HA group were retrieved from surgical reports. The S-GRAS score [16], encompassing sex, grade (according to the European Network for the Study of Adrenal Tumours (ENSAT) system), resection status, age, and tumour-related symptoms, was calculated for all ACC patients. The study was conducted in accordance with the principles of the Declaration of Helsinki, and institutional review board approval was obtained (EA1/394/20, 21 January 2021).

### Statistical analysis

Continuous variables are displayed as medians (range), categorical variables as frequencies. The Mann–Whitney *U* test was used for group comparison of metric variables, the chi-square or Fisher’s exact test for categorical variables. The Kaplan–Meier method was used to calculate overall survival (OS), defined as time between adrenalectomy and death and progression-free survival (PFS), defined as time between adrenalectomy and first postoperative progression in patients with adrenocortical carcinoma. Patients who did not reach the respective endpoints or were lost to follow-up were censored at the last follow-up visit. Patients without follow-up were excluded from the PFS and OS analyses. Survival rates were compared using log-rank tests. The prognostic value of variables was assessed with univariate Cox regression models. R1 and Rx categories were pooled to improve model stability due to small samples. To address multicollinearity and model instability, a multivariate analysis was performed using the Ridge Regression model with regularization parameter *λ* = 0.0174 applied for OS and *λ* = 0.4551 for PFS. The significance level was set to 0.05. Statistical analyses were performed using R version 024.12.0 + 467 (R Foundation for Statistical Computing, Vienna, Austria).

## Results

### Patient and adrenal tumour characteristics

During the observation period, a total of 97 patients underwent surgery for an adrenal tumour measuring at least 60 mm. While the most common indications for surgery included adrenocortical carcinomas (*n* = 33, 34%), pheochromocytomas and paragangliomas (*n* = 23, 23.7%), and adrenocortical adenomas (*n* = 19, 19.6%), other indications (*n* = 7) included single cases of an oncocytoma, sarcoma, fibroma, schwannoma, B-cell-Hodgkin lymphoma, ganglioneuroma, and haemangioma. Approximately one-quarter of the lesions infiltrated blood vessels (*n* = 25, 25.8%) while only a small portion of tumors (*n* = 5, 5.2%) exhibited infiltration into adjacent organs (kidney, liver, diaphragm, and lymph node). The characteristics of the study cohort are summarized in Table 1.

**Table 1:** Patient and tumour characteristics in surgically treated adrenal tumours measuring ≥ 60 mm (*n* = 97)

Patient and tumor characteristics		
Gender^a^	Female	51 (52.6%)
	Male	46 (47.4%)
Age (years)^b^		57 [18–86]
BMI (kg/m^2^)^b^ (n.a. = 3)		26.3 [17.9–78]
ASA^a^	1	6 (6.2%)
	2	40 (41.2%)
	3	48 (49.5%)
	4	3 (3.1%)
Tumour side^a^	Right	42 (43.3%)
	Left	55 (56.7%)
Tumour size (mm)^b^		84 [60–260]
Hormonal activity^a^ (n.a. = 1)		60 (62.5%)
Tumour entity^a^	Adrenocortical carcinoma	33 (34.0%)
	Adrenocortical adenoma	19 (19.6%)
	Adrenal metastasis	12 (12.4%)
	PPGL	23 (23.7%)
	Adrenal cyst	3 (3.1%)
	Other	7 (7.2%)
Lymph node metastases^a^		8 (8.2%)
Vascular infiltration^a^		25 (25.8%)
Infiltration of adjacent organs^a^		5 (5.2%)
Hormonal activity	Hormonally inactive^a^	37 (38%)
	Catecholamines^a^	20 (21%)
	Cortisol^a^	15 (16%)
	Aldosterone^a^	7 (7%)
	Androgen-related steroids^1^	4 (4%)
	Cortisol + Androgen-related steroids^a^	13 (13%)
	Cortisol + Catecholamines^a^	1 (1%)

*BMI* Body Mass Index, *ASA* American Association of Anesthesiologists, *n.a.* not available, *PPGL* phaeochromocytoma and paraganglioma, *ICU* Intensive Care Unit

^a^Count (percentage)

^b^Median [range]

### Intra- and postoperative outcomes of the study cohort

As shown in Table 2, 41 (42.3%) operations were conducted as MIA (38 via transperitoneal and 3 via retroperitoneoscopic approach), 40 (41.2%) as OA and 16 (16.5%) as HA. The transperitoneal laparoscopic approach was indicated due to suspected benign tumours (*n* = 18, 47%) or pheochromocytomas (*n* = 9, 24%). The retroperitoneoscopic approach was chosen in one case due to a suspected benign tumour (*n* = 1, 33.3%) and in another due to suspected adhesions after previous transabdominal operations (*n* = 1, 33.3%). In 11 patients (29%) operated on via transabdominal laparoscopy and in 1 patient (33.3%) via the retroperitoneoscopic approach, no explanation was provided in the operation report or discharge letter.

**Table 2:** Intra and postoperative outcomes after surgery for adrenal tumours measuring ≥ 60 mm (*n* = 97)

Intra and postoperative course		
Technique of operation^a^	Minimally invasive	41 (42.3%)
	Open surgery	40 (41.2%)
	Hybrid approach	16 (16.5%)
Duration of surgery (minutes)^b^ (n.a. = 4)		148 [52–825]
Multivisceral resection^a^		27 (27.8%)
Blood transfusion^a^		13 (13.4%)
ICU^a^		62 (63.9%)
Length of postoperative ICU stay (days)^b^		1 [0.5–38]
Length of postoperative hospital stay (days)^b^		6 [1–78]
Complications (any)^a^		25 (25.8%)
Type of complications^a^	Minor	20 (20.6%)
	Major	5 (5.2%)

*n.a.* not available, *ICU* Intensive Care Unit

^a^Count (percentage)

^b^Median [range]

Open surgery was performed due to suspected malignancy (*n* = 5, 12.5%), suspected organ infiltration (*n* = 2, 5%), planned multivisceral resection (*n* = 20, 50%), large tumour size (*n* = 8, 20%), suspected adhesions after abdominal operations (*n* = 1, 2.5%), and, in one patient (2.5%), due to heart insufficiency as a contraindication to laparoscopy. In 3 patients (7.5%) who underwent open surgery, no reason was documented in the operation report.

Planned hybrid adrenalectomy was chosen in 8 (89%) patients to achieve maximum tumour mobilisation laparoscopically before conversion, and in 1 (11%) patient because laparoscopic resection of a liver metastasis was planned. No specific reason for laparoscopic exploration was documented in the group of patients with unplanned conversion.

### Characteristics of patients undergoing hybrid adrenalectomy

As the study focused on the impact of conversion on intra- and postoperative outcomes, we provided a detailed description of the HA group (Table 3). Sixteen patients underwent surgery via a hybrid approach; in nine patients (56%), conversion was implemented as a planned strategic manoeuvre during the surgical procedure. The indications for surgery included adrenocortical carcinoma (*n* = 8), pheochromocytoma (*n* = 4), adrenal metastasis (*n* = 3) of a leiomyosarcoma, non-small cell lung cancer, hepatocellular carcinoma, and one case of sarcoma. In each patient, maximum tumour mobilization was reached laparoscopically before conversion. The reasons for conversion included adhesions to or infiltration of other organs (liver pancreas and/or kidney) or blood vessels and a poor anatomical overview due to the tumour size.

**Table 3:** Characteristics of patients who underwent hybrid adrenalectomy

Patient	Age	Gender	ASA	BMI (kg/m^2^)	Side	Size (mm)	Pathology	Reasons for conversion	Planned conversion
1	74	M	3	32,4	R	60	META	Infiltration of the liver	No
2	58	F	2	28.4	R	60	META	Infiltration of the vena cava inferior	No
3	59	M	3	26.5	L	95	PHEO	Poor anatomical overview due to tumour size	No
4	70	F	2	30.0	R	60	ACC	No laparoscopic preparation possible due to stretching of the vena cava inferior over the tumour	No
5	35	F	4	24.3	L	120	PHEO	Adhesion to pancreas and kidney; Poor anatomical overview due to tumour size	Yes
6	64	F	3	41.1	R	65	ACC	Elevated risk of capsule injury (thin capsule)	Yes
7	58	M	2	26.9	R	85	ACC	Adhesion to the kidney; Poor anatomical overview due to tumour size	Yes
8	39	M	2	25.7	L	75	PHEO	Adhesion to the kidney	No
9	72	M	3	31.0	L	130	ACC	Adhesion to the kidney	Yes
10	49	M	3	21.7	L	105	PHEO	Adhesion to pancreas	Yes
11	29	M	1	29.2	L	65	SARCOMA	Adhesion to kidney	No
12	61	F	2	29.7	R	67	ACC	Adhesion to the liver, elevated risk of capsule injury (soft tumour)	Yes
13	70	M	3	24.2	L	85	META	Poor anatomical overview due to tumour size	No
14	60	M	3	35.7	L	88	ACA	Adhesion to the kidney	Yes
15	49	M	3	28.1	L	130	ACC	Adhesion to the kidney	Yes
16	63	F	3	27.3	L	133	ACC	Poor anatomical overview due to tumour size	Yes

*M* male, *F* female, *ASA* American Association of Anesthesiologists classification, *BMI* body mass index, *R* right, *L* left, *META* adrenal metastasis, *PHEO* pheochromocytoma, *ACC* adrenocortical carcinoma, *ACA* adrenocortical adenoma

### Outcome according to the surgical technique used for adrenalectomy: comparison of the three groups

The number of female patients in the OA group was significantly greater than that in the MIA group (*n* = 28, 70% vs. *n* = 17, 41% *p* = 0.018). Moreover, no differences in age, BMI and ASA of the patients were found among the 3 groups, as presented in Table 4.

**Table 4:** Comparison of patient and tumour characteristics in patients undergoing minimally invasive (MIA), open (OA), and hybrid adrenalectomy (HA) for adrenal tumors measuring ≥ 60 mm (*n* = 97)

	MIA (*n* = 41)	OA (*n* = 40)	HA (*n* = 16)	*p* Value		
				MIA vs OA	MIA vs HA	OA vs HA
Gender^a^						
Female	17 (41.0%)	28 (70.0%)	6 (37.5%)	0.018	1	0.052
Male	24 (59.0%)	12 (30.0%)	10 (62.5%)			
Age (years)^b^	58 [28–86]	51.5 [18–84]	59.5 [29–74]	0.067	0.952	0.141
BMI (kg/m^2^)^b^	27 [17.9–42.3]	24.6 [18.2–78] (n.a. = 3)	28.25 [21.7–41]	0.140	0.241	0.025
ASA^a^						
1	4 (9.8%)	1 (2.5%)	1 (6.25%)	0.581	0.446	0.524
2	19 (46.3%)	16 (40.0%)	5 (31.25%)			
3	17 (41.5%)	22 (55.0%)	9 (56.25)			
4	1 (2.4%)	1 (2.5%)	1 (6.25%)			
Tumour side^a^						
Right	18 (43.9%)	18 (45.0%)	6 (37.5%)	1	0.888	0.831
Left	23 (56.1%)	22 (55.0%)	10 (62.5%)			
Tumour size (mm)^b^	70 [60–120]	100 [60–260]	85 [60–133]	< 0.001	0.293	0.031
Hormonal activity^a^	26 (63.4%)	23 (59.0%) (n.a. = 1)	11 (68.75%)	0.859	0.944	0.710
Tumour entity^a^						
ACC	3 (7.3%)	23 (57.5%)	7 (43.75%)	< 0.001	0.003	0.556
ACA	15 (36.6%)	3 (7.5%)	1 (6.25%)	< 0.001	0.023	1
MET	3 (7.3%)	6 (15.0%)	3 (18.75%)	0.482	0.338	0.700
PPGL	14 (34.1%)	5 (12.5%)	4 (25.0%)	0.019	0.542	0.250
CYST	2 (4.9%)	1 (2.5%)	0 (0%)	0.615	1	1
Other	4 (9.8%)	2 (5.0%)	1 (2.5%)	0.432	1	1
Lymph node metastases^a^	0 (0.0%)	8 (20%)	0 (0.0%)	0.002	1	0.089
Vascular infiltration^a^	3 (7.3%)	16 (40%)	6 (37.5%)	0.016	0.001	1
Infiltration of adjacent organs^a^	0 (0.0%)	4 (10%)	1 (6.3%)	0.054	0.281	1

*MIA* minimally invasive adrenalectomy, *OA* open adrenalectomy, *HA* hypbrid approach, *BMI* Body Mass Index, *ASA* American Association of Anesthesiologists, *n.a.* not available, *ACC* adrenocortical carcinoma, *ACA* adrenocortical adenoma, *MET* adrenal metastasis, *PPGL* phaeochromocytoma and paraganglioma, *CYST* adrenal cyst, *ICU* Intensive Care Unit

^a^Count (percentage)

^b^Median [range]

In the OA and HA groups, a significantly greater proportion of diagnoses of adrenocortical carcinoma were confirmed than in the MIA group (*n* = 23, 57.5% (*p* < 0.001) and *n* = 7, 43.75% (*p* = 0.003) versus *n* = 3, 7.3%, respectively). Conversely, minimally invasive adrenalectomies were performed more often for adrenal adenoma (*n* = 15, 36.6%) than open (*n* = 3, 7.5%) and hybrid adrenalectomies (*n* = 1, 6.25%); (*p* < 0.001 and *p* = 0.023, respectively). Phaeochromocytomas and paragangliomas were more often operated via MIA (*n* = 14, 34.1% than via OA (*n* = 5, 12.5%); (*p* = 0.019). Adrenal tumors operated via OA were significantly larger than those operated via MIA (median size 100 mm [range 60–260 mm] vs 70 mm [range 60–120 mm], *p* < 0.001). There were no statistical differences in tumour size between MIA and HA, as well as between HA and OA groups. Compared with tumors operated via laparoscopic or retroperitoneoscopic approaches, those operated via OA or HA had a greater rate of infiltration into neighbouring vessels (*n* = 16, 40% (*p* = 0.016) and *n* = 6, 37.5% (*p* = 0.001) vs *n* = 3, 7.5%, respectively). No differences in infiltration of adjacent organs were noted between the groups.

With respect to all other observed underlying conditions, no evidence of significantly different distributions between MIA, OA, and HA were identified. Moreover, the three groups did not differ in terms of the proportion of hormone-producing lesions or the side of the lesion.

Table 5 presents the group comparisons for the intra- and postoperative outcomes. Notably, regarding the duration of surgery, the MIA procedure lasted significantly shorter (median time 108.5 min [range 57–254 min]) than the OA and HA procedures did (median times 188 min [range 52–825] and 226.5 [range 136–466], respectively; *p* < 0.001 for both). At the same time, significantly less multivisceral resections were performed via MIA (*n* = 1, 2.4%) than OA (*n* = 20, 50%) and HA (*n* = 6, 37.5%), (*p* < 0.001 and *p* = 0.002, respectively).

**Table 5:** Comparison of intra- and postoperative outcomes in patients undergoing minimally invasive (MIA), open (OA), and hybrid adrenalectomy (HA) for adrenal tumors measuring ≥ 60 mm

	MIA (*n* = 41)	OA (*n* = 40)	HA (*n* = 16)	*p* Value		
				MIA vs OA	MIA vs HA	OA vs HA
Duration of surgery (minutes)^b^	108.5 [57–254] (n.a. = 1)	188 [52–825] (n.a. = 3)	226.5 [136–466]	< 0.001	< 0.001	0.102
Multivisceral resection^a^	1 (2.4%)	20 (50.0%)	6 (37.5%)	< 0.001	0.002	0.582
Blood transfusion^a^	2 (4.9%)	8 (20%)	3 (18.7%)	0.084	0.253	1
ICU stay^a^	16 (39%)	33 (82.5%)	13 (81.3%)	< 0.001	0.010	1
Length of postoperative ICU stay (days)^b^	1 [0.5–4]	1 [1–38]	1 [0.5–14]	0.237	0.417	0.903
Length of postoperative hospital stay (days)^b^	4 [1–13]	10 [3–78]	9 [4–40]	< 0.001	< 0.001	0.519
Complications (any)^a^	3 (7.3%)	15 (37.5%)	7 (43.7%)	0.003	0.004	0.897
Type of Complications^a^						0.6171
Major	0 (0.0%)	3 (7.5%)	2 (12.5%)	0.116	0.0752	
Minor	3 (7.3%)	12 (30%)	5 (31.25%)	0.011	0.032	

*MIA* minimally invasive adrenalectomy, *OA* open adrenalectomy, *HA* hybrid approach, *n.a.* not available, *ICU* Intensive Care Unit

No significant difference in the need for blood transfusion was detected among the groups. The occurrence of postoperative complications was lower in the MIA procedures than in the OA and HA procedures (*p* = 0.003 and *p* = 0.004, respectively). However, no differences in the rate of major complications were found. MIA patients were less frequently transferred to the ICU than OA and HA patients (*p* < 0.001 and *p* = 0.028, respectively) and had shorter hospital stays (*p* < 0.001 for both). When comparing the open and hybrid groups, no differences in patient and tumour characteristics as well as intra- or postoperative outcome parameters were demonstrated. Importantly, no patients died during the postoperative hospital stay.

^a^Count (percentage)

^b^Median [range]

In the study cohort, a total of 34 patients underwent surgery for adrenocortical carcinoma (ACC) measuring ≥ 60 mm. The characteristics of the patients are summarized in Table 6.

**Table 6:** Patient and tumour characteristics, along with intra- and postoperative outcomes after surgery for adrenocortical carcinoma measuring ≥ 60 mm (*n* = 34)

Patient and tumor characteristics		
Gender^a^	Female	22 (64.7%)
	Male	12 (35.3%)
Age (years)^b^		55 [18–77]
BMI (kg/m^2^)^b^ (n.a. = 2)		31 [23–78]
ASA^a^	1	2 (5.9%)
	2	11 (32.4%)
	3	20 (58.8%)
	4	1 (2.9%)
Localization of the adrenal tumour^a^	Right	17 (50.0%)
	Left	17 (50.0%)
Tumour size (mm)^b^		105 [65–260]
Hormonal activity^a^ (n.a. = 1)		24 (70.6%)
Ki67		17.5 [3–80]
R-status	R0	27 (79.4%)
	R1	4 (11.8%)
	Rx	3 (8.8%)
S-GRAS-Score	1	4 (11.8%)
	2	3 (8.8%)
	3	5 (14.7%)
	4	15 (44.1%)
	5	4 (11.8%)
	7	2 (5.9%)
	8	1 (2.9%)
Metastases to other organs at the time of operation		6 (17.6%)
Lymph node metastases^a^		6 (17.6%)
Vascular infiltration		15 (44.1%)
Infiltration of adjacent organs		4 (11.8%)
Intra- and postoperative course		
Technique^1^	Minimally invasive	4 (11.8%)
	Open surgery	23 (67.6%)
	Hybrid approach	7 (20.6%)
Duration of surgery (minutes)^b^ (n.a. = 2)		188 [73–825]
Multivisceral resection^a^	Yes	15 (44.1%)
Blood transfusion	Yes	8 (23.5%)
ICU stay^a^	Yes	26 (76.5%)
Length of postoperative ICU stay^b^		1 [0–38]
Length of postoperative hospital stay^b^		12.5 [3–78]
Complications (any)^a^	Yes	13 (18.1%)
Type of complications^a^	Minor	10 (29.4%)
	Major	3 (8.8%)

*n.a.* not available, *ICU* Intensive Care Unit

^a^Count (percentage)

^b^Median [range]

Complete resection (R0) was achieved in 27 ACC patients (79.4%), while 4 patients (11.8%) had microscopic residual disease (R1), including two operated via HA and two via OA. Additionally, 3 patients (8.8%) had an unknown resection status (Rx), all of whom underwent OA.

**Fig. 1:**
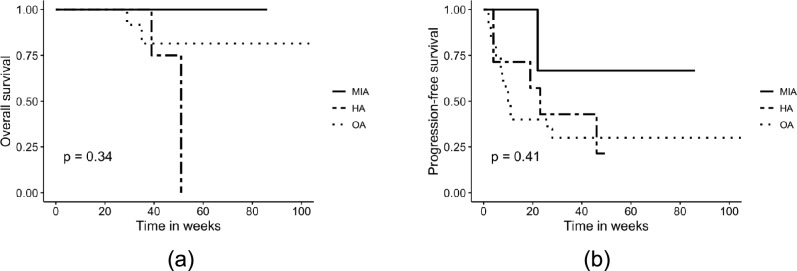
Comparison of **a** overall (OS) and **b** progression-free survival (PFS) in patients undergoing a minimally invasive (MIA), open (OA) and hybrid adrenalectomy (HA) for adrenocortical carcinoma. Survival rates were compared using log-rank test. No statistically significant differences were found between the groups

For three patients with ACC who underwent open surgery, no follow-up data were available. These patients were excluded from the survival analysis. Among patients with ACC included in the survival analysis, the median follow-up time was 37 months [range: 3–154 months], and the median progression-free survival was 19 months [range: 2–154 months]. Survival analysis showed no statistically significant differences in overall survival (OS) and progression-free survival (PFS) between surgical techniques (log-rank *p* > 0.05), as shown in Fig. 1. A pairwise comparison of OS and PFS between hybrid and open adrenalectomy showed no significant differences (*p* = 0.31 and *p* = 0.84, respectively).

In the univariate Cox regression analysis of the five S-GRAS score components in ACC, tumour stage (according to ENSAT classification) was a significant predictor of overall survival (OS) (HR = 4.66, *p* = 0.041) and progression-free survival (PFS) (HR = 2.56, *p* = 0.004) (see Supplementary Tables 1 and 2, respectively). The Ki-67 index was significantly associated with PFS (HR = 1.03, *p* = 0.004) but not with OS (HR = 1.06, *p* = 0.081), while tumour size, patient’s age at surgery, and tumour resection status had no significant effect on OS or PFS of ACC patients.

As presented in Supplementary Tables 3 and 4, the univariate cox regression analysis of the S-GRAS score, ASA classification, and surgical technique identified S-GRAS score as predictive of OS (HR = 5.79, *p* = 0.0257 and PFS (HR = 2.48; *p* = 0.0113). Both, ASA classification and the surgical method had no significant effect on overall and progression-free survival. However, wide confidence intervals demonstrate the model’s instability. The Ridge Regression model stabilized estimates and confirmed the S-GRAS score as an independent predictor of OS (HR = 6.00) and PFS (HR = 1.50). ASA classification and surgical technique did not significantly impact survival outcomes. The results of the Ridge regression analysis for S-GRAS score, ASA classification, and surgical technique in relation to survival are presented in the Supplementary Tables 5 and Table 6.

Patients with the lowest S-GRAS scores (1–3) had a longer PFS than those with S-GRAS scores of 4–5 (*p* = 0.016), but not a significantly longer OS (*p* = 0.14). Compared to patients with an S-GRAS score of 6–8, those with the lowest S-GRAS scores showed favorable PFS (*p* = 0.026) and OS (*p* = 0.003). No significant difference in OS or PFS was observed between patients with S-GRAS scores of 4–5 and 6–8. A detailed analysis of the impact of the S-GRAS score on survival is presented in Supplementary Fig. 1.

## Discussion

This study primarily aimed to analyse the outcomes of hybrid adrenalectomy (HA**)** for large adrenal tumors (≥ 60 mm), comparing it to minimally invasive adrenalectomy (MIA) and open adrenalectomy (OA). MIA offered shorter operative times, reduced minor complication rates, and shorter hospital stays and was predominantly utilized for smaller and less invasive tumors. In contrast, OA and HA were more frequently performed for more aggressive tumors, including adrenocortical carcinomas (ACC), and for cases requiring multivisceral resections. Importantly, no significant differences in intra- and postoperative outcomes were observed between OA and HA in our patients, nor did we discover significant differences in overall survival (OS) or progression-free survival (PFS) in patients with ACC treated with different surgical techniques. These findings support a tailored approach for large adrenal tumours. Preoperative assessment and individualized surgical procedure based on tumour characteristics therefore seem possible and mandatory.

With increasing experience in minimally invasive adrenal surgery, the indications for laparoscopic adrenalectomy have broadened [6, 8, 17–20]. Reported conversion rates vary, from none [21] to 20% [22]. In our cohort, the conversion rate for adrenal masses measuring ≥ 60 mm was 28%. Planned conversions because of inadequate anatomical overview, adhesions to surrounding structures or infiltration of adjacent organs accounted for 15.8%, while intraoperative conversion in primarily questionable cases was indicated to prevent tumour rupture or other unnecessary complications in 12.3% of cases. Contrary to the findings reported by Higashihara et al., where bleeding caused 45% of conversion [9], none of the conversions in our cohort were performed due to bleeding. The need for possible conversion should be anticipated as part of the surgical strategy for large and complex tumors.

A few studies have compared converted adrenalectomies with minimally invasive procedures in terms of intraoperative and immediate postoperative outcomes. Schweitzer et al*.* reported that tumour size greater than 60 mm was associated with conversion to open surgery [23]. However, Gaujoux et al. found no association between tumour size and conversion in their cohort [13]. Notably, in their study, tumours larger than 120 mm were primarily approached via open surgery. In addition, they identified conversion to open surgery as an independent predictive factor for medical complications, including pulmonary and thromboembolic events as well as organ failure, but not for surgical complications [13]. Similarly, Schweitzer et al. reported that conversion was associated with a postoperative Clavien-Dindo grade II–V morbidity in patients undergoing surgical treatment for pheochromocytoma [23]. No distinction was made between medical and surgical complications in their analysis. In our cohort, conversion was associated with a higher rate of minor complications but did not lead to an increase in major complications compared to MIA.

In line with the findings of Schweitzer et al., our patient cohort also showed a longer hospital stay following conversion to open adrenalectomy compared to minimally invasive surgery [23]. Additionally, consistent with the results of Gaujoux et al. [13] and Higashihara et al. [9], conversion in our cohort was associated with prolonged operative time. However, tumors operated via HA were more frequently malignant and demonstrated greater vascular infiltration and lymph node involvement. Thus, as possible explanations for the prolonged operation time, surgical complexity and radicality can be assumed. Given these factors, a more relevant question is whether conversion leads to worse postoperative outcomes compared to direct open surgery. More specifically, it is worth considering whether certain intra- and postoperative complications could be avoided by opting directly for open surgery rather than beginning with a minimally invasive approach while anticipating the possibility of conversion. Importantly, in our patient cohort, no statistically significant differences were observed between HA and OA in this regard.

While combining laparoscopic and open approaches in one operation has been studied in pancreatic surgery [24, 25], data on the hybrid approach in adrenalectomy are scarce. The strategic use of conversion may offer a balanced approach by combining the benefits of minimally invasive techniques with the safety of open surgery in challenging cases [14]. Laparoscopy not only provides reduced postoperative pain and shorter hospital stays [8], but also offers technical benefits in adrenal surgery given the anatomic location. The distal subdiaphragmatic region can be reached more easily via laparoscopy than via open surgery. Thus, mobilization of the spleen or liver and parts of the adrenal tumor laparoscopically may facilitate the next surgical steps conducted via the open approach. The open approach allows for easier management of vascular structures, identification of tumor infiltration and, therefore, a reduced risk of incomplete tumour resection [26]. The potential advantage of the hybrid approach in allowing for a smaller incision compared to primary open surgery has yet to be studied.

While none of the adrenalectomies for tumors larger than 60 mm were performed via the robotic approach (RA) during the study period at our center, the method has been increasingly applied in other centers [27], particularly in large tumors [28]. Studies suggest that RA offers advantages in specific clinical scenarios, including shorter operative times for tumors ≥ 6 mm, patients with high BMI, and those with previous abdominal surgery [29, 30]. RA is also associated with shorter hospital stays [31, 32]. Interestingly, the need for conversion decreases with tumor size in RA compared to LA [33, 34]. While conversion remains safe when necessary, RA represents an increasingly preferred option for large adrenal tumors, particularly where resources and expertise are available. Importantly, while LA remains more cost-effective overall, this difference decreases with increasing tumor size, making RA comparatively more efficient for tumors ≥ 40 mm [35]. Moreover, recent institutional data suggest that RA may lead to lower overall costs due to shorter hospital stays, despite higher disposable expenses [32]. Nevertheless, access to robotic platforms remains limited in many centers [28], highlighting the continued relevance of discussing minimally invasive, hybrid and open approaches.

Unlike the findings of Delozier et al*.* [10], our results showed that conversion did not negatively affect survival outcomes in ACC. Both the S-GRAS score and ENSAT classification emerged as significant predictors of OS and PFS, while the choice of surgical method—whether MIA, OA, or HA—did not significantly impact survival outcomes. Thus, in our patients with ACC, survival outcomes were predominantly influenced by tumour biology and stage rather than the choice of surgical technique.

Our data highlight the critical importance of careful preoperative assessment and a tailored surgical approach based on tumour characteristics and the individual patient’s clinical status. Conversion to an open approach should be considered as a strategic maneuver to optimize oncologic outcomes [3, 4], rather than being perceived as a surgical failure. Future studies should explore whether a proactive, strategy-driven approach to conversion can further improve outcomes in complex adrenal surgeries, particularly in cases of malignancy.

This study has several limitations that should be considered when interpreting the results. First, it was conducted over an extended period at a single tertiary referral center for endocrine surgery, where all adrenalectomies were performed by highly experienced visceral surgeons. This high level of surgical expertise and the specialized setting may not reflect broader clinical practice, potentially limiting the generalizability of our findings to other institutions or surgical teams with different levels of experience and resources. However, complex adrenal surgeries, especially in cases suspected of malignancy, should be referred to specialized centers [4]. Second, adrenal tumors measuring at least 60 mm are relatively rare, resulting in a limited cohort size with heterogeneous adrenal pathologies. Lastly, our study was retrospective in nature, and prospective studies with larger patient cohorts are needed to validate our findings and explore the impact of conversion on short-term surgical and medical, as well as long-term oncologic outcomes in cases of adrenal malignancy.

## Conclusions

Our study highlights the importance of an individualized surgical approach for large adrenal tumors (≥ 60 mm) that considers both tumor-specific and patient-specific factors as well as the surgeon’s expertise. While minimally invasive adrenalectomy offers advantages such as shorter operative times and reduced complication rates, the use of open and hybrid approaches is justified in complex cases, particularly when addressing aggressive or large tumors. Importantly, conversion from minimally invasive to open surgery should not be regarded as a complication but rather as a strategic decision that enhances patient safety and optimizes oncologic outcomes. Our results indicate that conversion did not significantly impact overall or progression-free survival in adrenocortical carcinoma patients. Instead, the S-GRAS score and ENSAT classification were the primary predictors of survival, underscoring the need for a robust preoperative assessment and a tailored surgical approach. Further prospective studies are necessary to evaluate and compare the roles of laparoscopic, hybrid, open, and robotic adrenalectomy in improving intraoperative safety, oncologic outcomes and cost-effectiveness in surgical management of large and complex adrenal tumors.

## Supplementary Information

Below is the link to the electronic supplementary material.

Supplementary file1 (DOCX 356 KB)

## Data Availability

The datasets generated during and analyzed during the current study are not publicly available due to reasons of sensitivity and are only available from the corresponding author on reasonable request.
