# Supplementary material for: Tailored surgery for large adrenal tumors: the minimally invasive to open (hybrid) approach
Source: Updates Surg. 2025 Aug 29;78(1):385–95. doi: 10.1007/s13304-025-02388-7 (PMC12909437; doi:10.1007/s13304-025-02388-7)
Supplement: Supplementary file 1 — Supplementary file1 (DOCX 356 KB) [file 13304_2025_2388_MOESM1_ESM.docx]

**Supplementary material**

**Supplementary Table 1.** Results of a univariate cox regression analysis of the five S-GRAS score components in adrenocortical carcinoma in relation to overall survival.

| **Variable** | **HR** | **95% CI** | **p-value** |
| --- | --- | --- | --- |
| Age at surgery | 1.06 | 0.97–1.16 | 0.217 |
| Tumour size | 0.99 | 0.96–1.01 | 0.308 |
| Tumour stage (ENSAT) | 4.66 | 1.06–20.42 | **0.041** |
| R Classification (R0 vs R1 and Rx) | 5.55 | 0.76–40.27 | 0.09 |
| Ki-67 Index | 1.06 | 0.99–1.13 | 0.081 |

HR, Hazard Ratio; CI, Confidence Interval; ENSAT, European Network for the Study of Adrenal Tumours; R, residual tumour.

**Supplementary Table 2.** Results of a univariate cox regression analysis of the five S-GRAS score components in adrenocortical carcinoma in relation to progression-free survival.

| **Variable** | **HR** | **95% CI** | **p-value** |
| --- | --- | --- | --- |
| Age at surgery | 0.99 | 0.97–1.02 | 0.465 |
| Tumour size | 1.00 | 0.99–1.01 | 0.501 |
| Tumour stage (ENSAT) | 2.56 | 1.34–4.88 | **0.004** |
| R Classification (R0 vs. Rx and R1) | 1.62 | 0.58–4.49 | 0.353 |
| Ki-67 Index | 1.03 | 1.01–1.05 | **0.004** |

HR, Hazard Ratio; CI, Confidence Interval; ENSAT, European Network for the Study of Adrenal Tumours; R, residual tumour.

**Supplementary Table 3.** Results of a univariate cox regression analysis of the S-GRAS Score, ASA Classification, and surgical technique in relation to overall survival in adrenocortical carcinoma.

| **Variable** | **HR** | **95% CI** | **p-value** |
| --- | --- | --- | --- |
| S-GRAS Score | 5.79 | 1.238 – 27.1 | **0.026** |
| ASA2 vs ASA1 | 0.87 | 0 – ∞ | 1.00 |
| ASA3 vs ASA1 | 1.57e+09 | 0 – ∞ | 1.00 |
| MIA vs HA | 5.7e+08 | 0 – ∞ | 0.999 |
| OA vs HA | 2.07e+08 | 0 – ∞ | 0.999 |

HR, Hazard Ratio; SE, Standard Error; CI, Confidence Interval; S-GRAS, sex, grading, resection status, age and tumour- or hormone-related symptoms; ASA, American Association of Anesthesiologists; MIA, minimally invasive adrenalectomy; HA, hybrid adrenalectomy; OA, open adrenalectomy

**Supplementary Table 4.** Results of a univariate cox regression analysis of the S-GRAS Score, ASA Classification, and surgical technique in relation to progression-free survival in adrenocortical carcinoma.

| **Variable** | **HR** | **95% CI** | **p-value** |
| --- | --- | --- | --- |
| S-GRAS Score | 2.48 | 1.228 – 5.004 | **0.011** |
| ASA2 vs ASA1 | 0.545 | 0.056 – 5.288 | 0.601 |
| ASA3 vs ASA1 | 3.006 | 0.394 – 22.964 | 0.289 |
| MIA vs HA | 3.269 | 0.381 – 28.04 | 0.280 |
| OA vs HA | 3.508 | 0.458 – 26.87 | 0.227 |

HR, Hazard Ratio; SE, Standard Error; CI, Confidence Interval; S-GRAS, sex, grading, resection status, age and tumour- or hormone-related symptoms; ASA, American Association of Anesthesiologists; MIA, minimally invasive adrenalectomy; HA, hybrid adrenalectomy; OA, open adrenalectomy

**Supplementary Table 5.**

Multivariate Ridge regression analysis (λ = 0.0174) for S-GRAS Score, ASA Classification, and surgical technique in relation to overall survival.

| **Variable** | **Coefficient (β)** | **HR (exp(β))** |
| --- | --- | --- |
| S-GRAS Score | 1.7913 | 6.00 |
| ASA2 vs ASA1 | -1.1642 | 0.31 |
| ASA3 vs ASA1 | 1.1915 | 3.29 |
| MIA vs HA | -0.5035 | 0.60 |
| OA vs HA | 0.7437 | 2.10 |

HR, Hazard Ratio; SE, Standard Error; CI, Confidence Interval; S-GRAS, sex, grading, resection status, age and tumour- or hormone-related symptoms; ASA, American Association of Anesthesiologists; MIA, minimally invasive adrenalectomy; HA, hybrid adrenalectomy; OA, open adrenalectomy

**Supplementary Table 6.** Ridge regression analysis (**λ = 0.4551)** for S-GRAS Score, ASA Classification, and surgical technique in relation to progression-free survival.

| **Variable** | **Coefficient (β)** | **HR (exp(β))** |
| --- | --- | --- |
| S-GRAS Score | 0.4074 | 1.50 |
| ASA2 vs ASA1 | -0.4588 | 0.63 |
| ASA3 vs ASA1 | 0.4552 | 1.58 |
| MIA vs HA | -0.0759 | 0.93 |
| OA vs HA | 0.2086 | 1.23 |

HR, Hazard Ratio; S-GRAS, sex, grading, resection status, age and tumour- or hormone-related symptoms; ASA, American Association of Anesthesiologists; MIA, minimally invasive adrenalectomy; HA, hybrid adrenalectomy; OA, open adrenalectomy


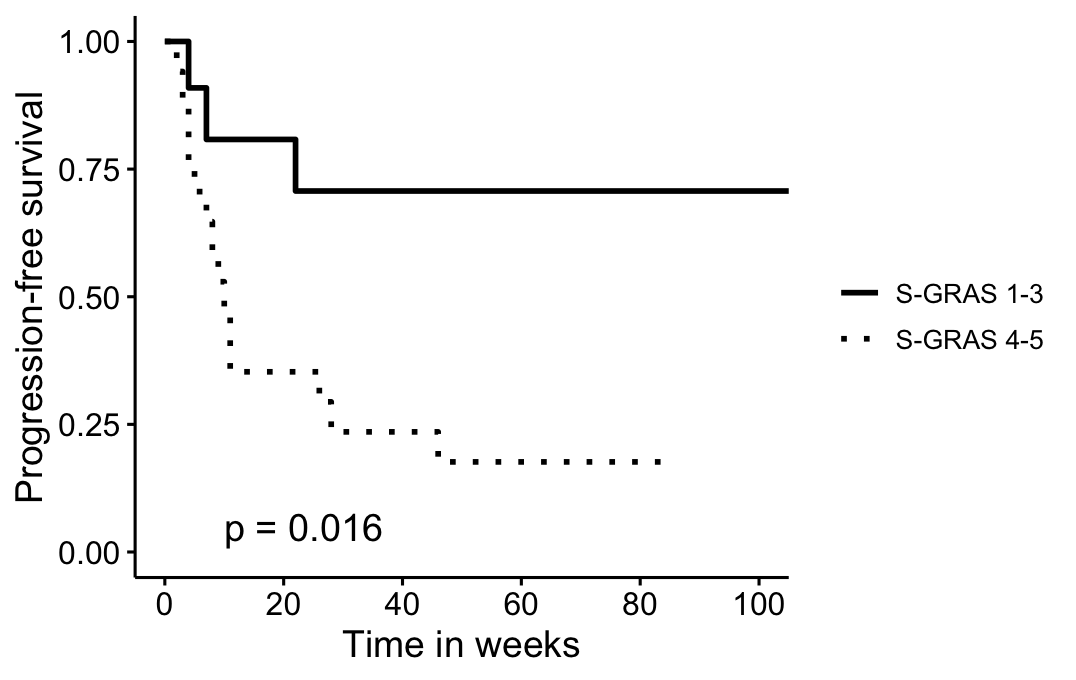

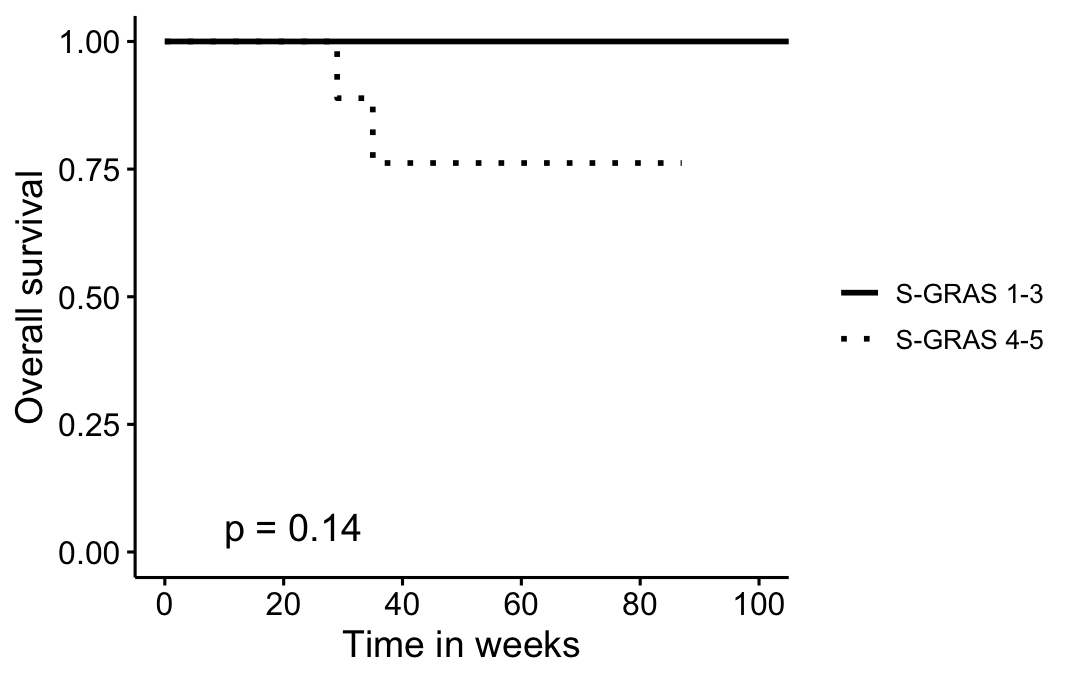


(a) (b)


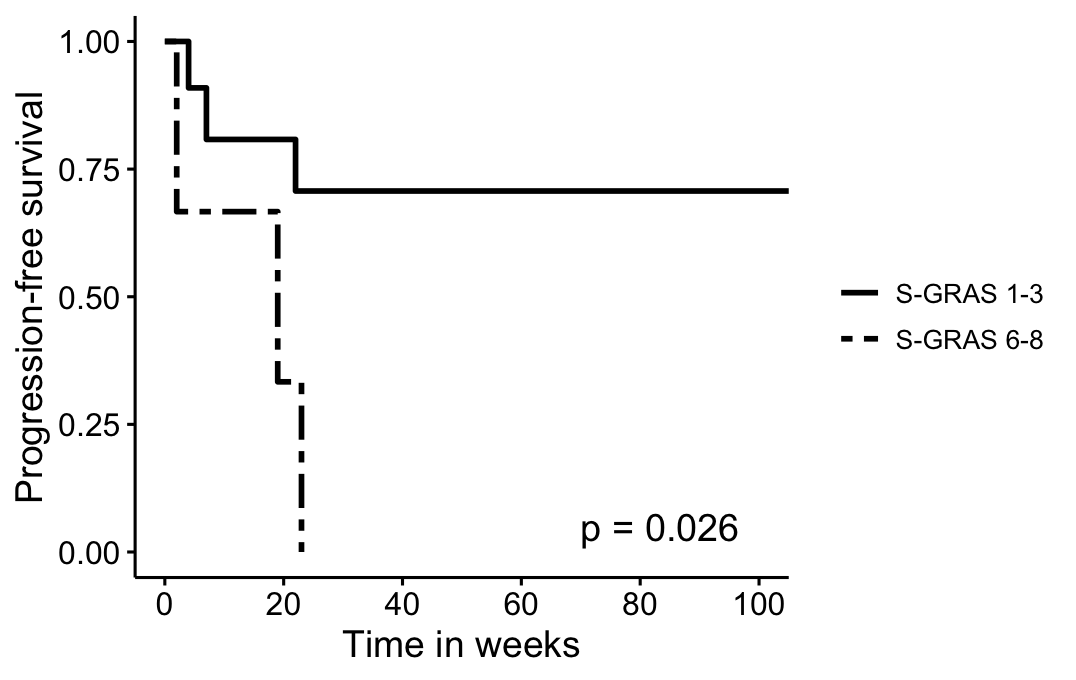

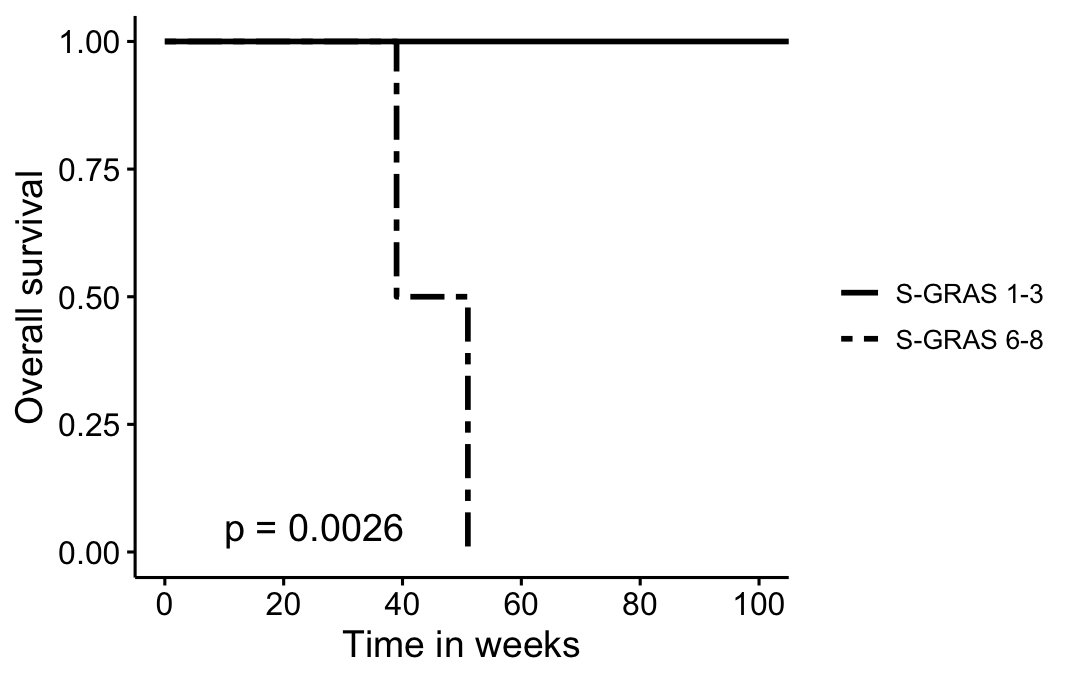


(c) (d)


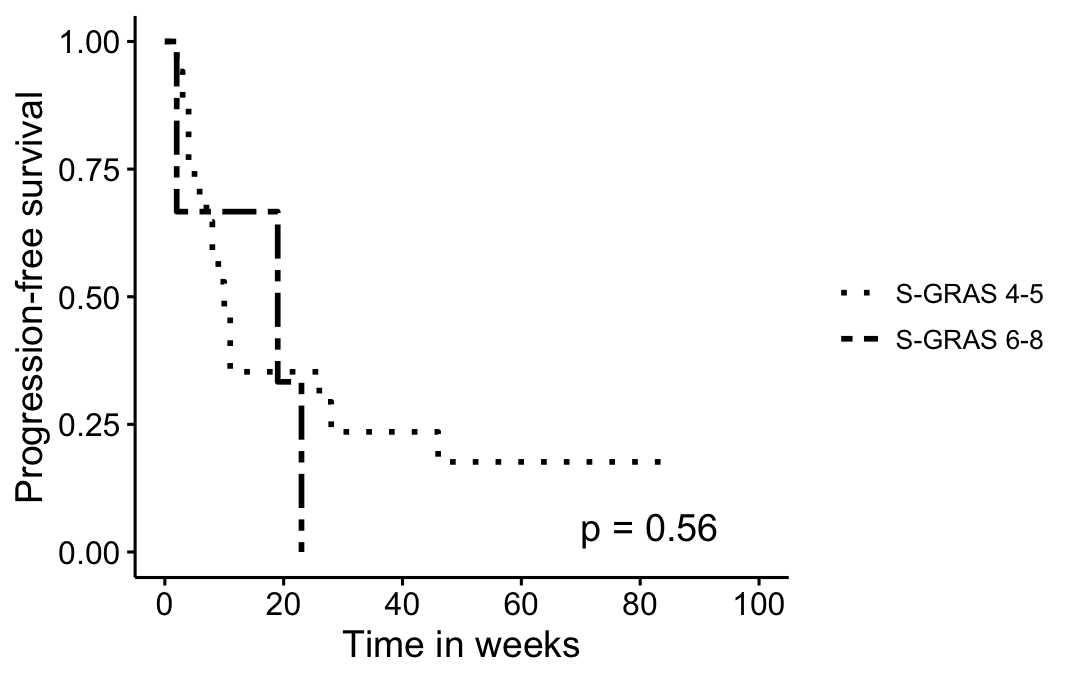

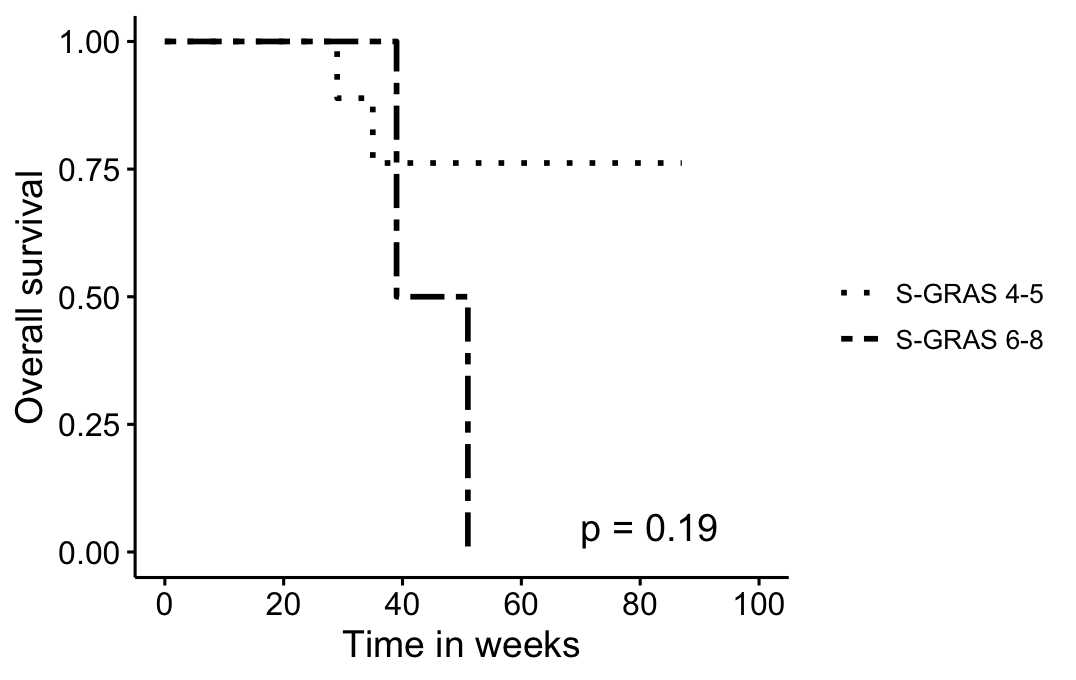


(e) (f)

**Supplementary Figure 1.** Pairwise comparison of overall (OS) and progression-free survival (PFS) in patients undergoing an adrenalectomy for adrenocortical carcinoma based on the S-GRAS Score. a. OS and b. PFS in patients with an S-GRAS Score 1-3 and 4-5. c. OS and d. PFS in patients with an S-GRAS Score 1-3 and 6-8; e. OS and f. PFS in patients with an S-GRAS Score 4-5 and 6-8. Survival rates were compared using log-rank test.
